# Prospective study of the effects of isotretinoin and vitamin D levels on severe acne vulgaris

**DOI:** 10.55730/1300-0144.5742

**Published:** 2023-09-20

**Authors:** Ramadan Sayed HUSSEIN, Salman Bin DAYEL, Othman ABAHUSSEIN

**Affiliations:** Department of Dermatology, College of Medicine, Prince Sattam bin Abdulaziz University, Al-Kharj, Saudi Arabia

**Keywords:** Acne vulgaris, vitamin D, isotretinoin

## Abstract

**Background/aim:**

Acne vulgaris (AV) is a common inflammatory skin condition associated with psychological and social distress. Its pathogenesis involves factors such as sebaceous hypersecretion and *Cutibacterium acnes* colonization. Vitamin D plays a crucial role in the pathogenesis of various inflammatory skin disorders, including AV, due to its immunomodulatory effects and involvement in keratinocyte growth and maturity. However, adequate sun exposure is required for optimal vitamin D synthesis. Isotretinoin (IOS), a vitamin A derivative, is a commonly used medication for severe acne, acting by binding to retinoid receptors. It can also form heterodimers with vitamin D receptors, potentially increasing vitamin D catabolism. Previous studies examining the impact of oral IOS on serum vitamin D levels have yielded inconsistent results. Therefore, this study aimed to assess changes in 25-hydroxy (OH) vitamin D serum levels in individuals with severe AV before and after IOS treatment.

**Materials and methods:**

One hundred patients with severe AV were enrolled, each receiving 0.75 mg/kg IOS treatment daily for 4 months. Serum 25 OH vitamin D levels were measured before, during, and after treatment.

**Results:**

This study found a significant increase in serum 25 OH vitamin D levels among patients with severe AV following IOS treatment (p < 0.001).

**Conclusion:**

This study suggests that AV may negatively impact vitamin D synthesis, but IOS treatment appears to raise vitamin D serum levels in individuals with severe AV. Further research is needed to confirm the potential relationship between AV and vitamin D levels.

## 1. Introduction

Acne vulgaris (AV), a common and distressing skin condition, predominantly affects adolescents [1]. Its pathogenesis is multifaceted, involving factors such as sebaceous hypersecretion, *Cutibacterium acnes* colonization, and intricate inflammatory and immunological responses [2]. Among these, androgen stimulation, insulin-like growth factor 1 (IGF-1), and vitamin D contribute to sebaceous gland activity, fostering a lipid-rich, anaerobic environment conducive to *C. acnes* growth [3]. Toll-like receptors and inflammation further contribute to follicular hyperkeratinization [4].

Vitamin D, pivotal for bone health and immune function, also impacts skin physiology and is synthesized through sunlight exposure [5]. Acting as an immunomodulator via vitamin D receptors (VDRs), it affects various immune cells, including dendritic cells, influencing immune responses [6]. Moreover, vitamin D regulates Th17 cell-mediated production of proinflammatory cytokines and sebocyte functions, suggesting a role in acne pathogenesis [7–9].

While vitamin D has known roles in health conditions including osteoporosis and cardiovascular disorders, its specific role in inflammatory skin disorders such as acne, attributed to its immunomodulatory effects and effects on the growth and differentiation of keratinocytes, is still the subject of ongoing research [9].

AV can be treated with diverse methods, including systemic therapies like isotretinoin (ISO), a synthetic retinoid derivative of vitamin A [10]. ISO is highly effective, reducing acne lesions through various mechanisms [11]. However, it poses potential adverse effects, including hepatitis, teratogenicity, skin dryness, psychological concerns, and cerebral ischemia [11].

Furthermore, the use of ISO has raised questions regarding its impact on vitamin D levels, as it can form heterodimers with VDRs and potentially affect vitamin D metabolism. Previous studies have yielded conflicting results regarding the effects of ISO on vitamin D levels in acne patients. Consequently, this study aims to investigate the influence of ISO treatment on serum vitamin D levels in individuals with severe AV, shedding light on its clinical implications.

## 2. Materials and methods

### 2.1. Study design

This prospective observational study was conducted at a dermatology outpatient clinic during the summer months.

### 2.2. Participants

One hundred participants were recruited based on the following detailed inclusion and exclusion criteria:

Inclusion criteria:

1. Participants were required to have a clinical diagnosis of severe AV as determined by the Global Acne Grading System (GAGS).
2. Participants were aged between 18 and 30 years.
3. Participants had skin types IV (moderate brown) and V (dark brown) according to the Fitzpatrick scale.
4. Both male and unmarried female participants were considered.
5. Written informed consent was obtained from all eligible participants.

Exclusion criteria:

1. Individuals with a history of liver or kidney disease, which might affect vitamin D metabolism, were excluded.
2. Participants taking vitamin D supplements or medications known to influence vitamin D levels were excluded.
3. Individuals on medications with known effects on vitamin D metabolism or absorption were not included.
4. Pregnant or breastfeeding individuals were excluded due to potential safety concerns related to IOS treatment.
5. Participants with severe dermatological conditions other than AV, which could confound the study results, were not included.
6. Individuals unable or unwilling to adhere to the study protocol or follow-up visits were excluded.
7. Participants were instructed to apply sunscreen with appropriate sun protection factors before sun exposure. Those who did not comply with this instruction were excluded to minimize the influence of UVB exposure on vitamin D synthesis.

### 2.3. Intervention

The treatment protocol for all participants consisted of taking 0.75 mg/kg IOS daily for a period of 4 months. Before sun exposure, all participants were directed to apply sunscreens with appropriate sun protection factors. The patients were closely monitored during treatment for any harmful effects.

### 2.4. Data collection

The researchers collected data by measuring the levels of 25-hydroxy (OH) vitamin D in blood serum at three time points: before starting treatment, during treatment, and after treatment. Samples were collected in the same season to minimize the impact of seasonal variations. Blood samples were collected via venipuncture and stored at a temperature of −80 °C until they were ready to be analyzed. Serum levels of 25 OH vitamin D were measured using an ELISA kit.

### 2.5. Sample size determination

The researchers used G*Power software version 3.1.9.4 to determine the minimum sample size required for the study. The estimate was based on previous studies and indicated that 80 patients would need to be included to detect a significant difference in serum vitamin D levels, with power of 0.80 and a significance level of 0.05. Consequently, 100 patients were enrolled to ensure adequate statistical power.

### 2.6. Statistical analysis

Data analysis was performed using IBM SPSS Statistics 25.0 (IBM Corp., Armonk, NY, USA). Descriptive statistics were employed to summarize the data. The paired t-test was utilized to assess changes in 25 OH vitamin D serum levels before and after ISO treatment.

### 2.7. Ethical considerations

The investigation was carried out in accordance with the ethical principles outlined in the Declaration of Helsinki, and all patients provided written informed consent. The study protocol was approved by the institutional review board of Prince Sattam bin Abdulaziz University (19/12/2022, No. SCBR-99/2022).

## 3. Results

Demographic characteristics of the study participants are provided in Table 1. This study included 100 patients who had severe AV. The patients had an average age of 22.8 years with a standard deviation of 5.6 years. Among the patients, 67% were female.

**Figure 1:**
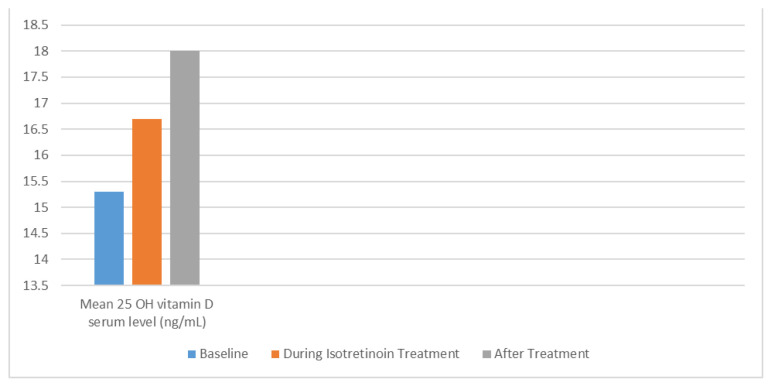
Changes in Serum 25 OH Vitamin D Levels During ISO Treatment.

Initially, the average level of 25 OH vitamin D in the patients’ serum was 15.3 ng/mL with a standard deviation of 3.2 ng/mL (Table 2). After receiving IOS treatment, the average serum level of 25 OH vitamin D increased significantly to 16.7 ng/mL with a standard deviation of 3.8 ng/mL (p < 0.001). Furthermore, after treatment completion, the mean serum level of 25 OH vitamin D further increased to 18.5 ng/mL with a standard deviation of 4.1 ng/mL (p < 0.001) (Figure 1). However, no significant relationship was found between the severity of AV and baseline vitamin D levels (p = 0.325). No significant adverse effects were reported during or after the treatment as detailed in Table 2.

**Figure 2:**
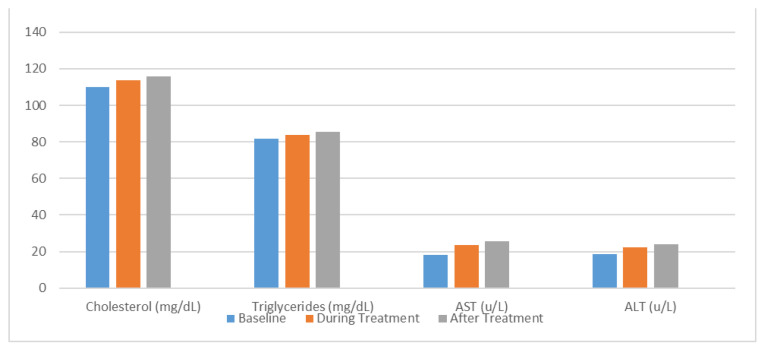
Changes in serum lipid profile and liver enzymes during ISO treatment.

Other parameters were also measured, including cholesterol, triglycerides, aspartate transaminase, and alanine transaminase (Figure 2). The levels of these parameters increased slightly during and after IOS treatment, but the differences were not statistically significant (Table 3).

## 4. Discussion

AV is a prevalent inflammatory skin condition that affects a significant proportion of the population worldwide. Its pathogenesis involves various factors such as sebaceous hypersecretion, follicular colonization by *C. acnes*, and inflammatory and immunological responses. Research has shown that vitamin D plays a vital role in the pathogenesis of many inflammatory skin conditions, such as AV [12]. Vitamin D is a potent immunomodulator and has been shown to regulate the growth and maturation of keratinocytes, which are key players in the development of AV [13].

IOS is a synthetic retinoid that is commonly prescribed to treat severe AV. It works by reducing sebum production, inhibiting follicular colonization by *C. acnes*, and suppressing the inflammatory and immune responses that are involved in the pathogenesis of AV [14]. Additionally, IOS has been shown to bind to retinoid receptors, which can combine with VDRs to form heterodimers. This process could lead to a reduction in vitamin D levels by increasing catabolism [15].

The impact of IOS on serum vitamin D levels has produced inconsistent findings in previous research [16,17]. This study focused on examining the 25 OH vitamin D levels of patients with severe AV before and after they received IOS treatment. The results showed a notable rise in 25 OH vitamin D levels in the patients’ serum after they received IOS treatment for severe AV.

Our research results align with some prior studies that also showed a rise in serum vitamin D levels following the administration of IOS treatment [17,18]. However, other research has found no significant changes in serum vitamin D levels after IOS treatment [19,20]. These differences in findings between studies could be due to variations in factors such as the study’s design, sample size, duration of treatment, and baseline vitamin D levels.

The precise mechanism by which IOS impacts vitamin D levels remains unclear. One possible explanation is that IOS may reduce the expression of 24-hydroxylase, which is responsible for the catabolism of vitamin D [17]. Another possible explanation is that IOS may alter the expression of vitamin D binding protein and increase the absorption of vitamin D from the intestines [21].

Our study has some limitations that should be acknowledged. First, we did not measure other markers of vitamin D metabolism, such as parathyroid hormone or calcium levels. Second, we did not assess the long-term impact of IOS on vitamin D levels. Third, we did not evaluate the impact of IOS on the levels of vitamin D in patients with mild to moderate AV.

In conclusion, our findings suggest that IOS treatment may increase 25 OH vitamin D levels in patients suffering from severe AV. This may have important implications for the management of AV and the potential association between vitamin D and its onset. Additional research is needed to confirm our results and reveal the mechanisms that govern the effect of IOS on vitamin D levels. Furthermore, it may be advisable to consider monitoring vitamin D levels in individuals undergoing IOS treatment for AV, especially those who are at risk of vitamin D deficiency.

## Figures and Tables

**Table 1:** Demographic characteristics of study participants.

Characteristics	Number (%) or mean ± SD
Age, years	22.8 ± 5.6
Sex, female	67 (67%)
Skin type, IV/V	100 (100%)

**Table 2:** Mean 25 OH vitamin D serum levels (ng/mL) at baseline and during and after treatment.

Parameters	Baseline	During isotretinoin treatment	After treatment
Number of patients	100	100	100
Mean age, years	22.8	N/A	N/A
Sex, female, %	67	N/A	N/A
Mean 25 OH vitamin D serum level, ng/mL	15.3 ± 3.2	16.7 ± 3.8*	18.5 ± 4.1*
r	0.325	N/A	N/A
Adverse effects	None reported	N/A	N/A

r: Correlation between baseline vitamin D levels and severity of acne vulgaris (p-value).

*: Significant difference between baseline and during/after treatment; IOS treatment was highly statistically significant at p < 0.001.

**Table 3:** Comparison of lipid profiles and liver enzymes before and after ISO treatment.

Parameters	Baseline	During treatment	After treatment
Cholesterol, mg/dL	110.07 ± 21.06	113.63 ± 21.41	115.67 ± 22.41
Triglycerides, mg/dL	81.94 ± 20.75	83.63 ± 22.04	85.63 ± 20.04
AST, U/L	18.32 ± 7.18	23.55 ± 9.53	25.54 ± 9.43
ALT, U/L	18.60 ± 6.84	22.52 ± 6.12	23.82 ± 5.32

AST: Aspartate transaminase; ALT: alanine transaminase.
